# Experimental Optimization Study on Pumping Pipeline Transportation Performance of Pure Gangue Slurry Filling Material

**DOI:** 10.3390/ma18204788

**Published:** 2025-10-20

**Authors:** Yingbo Wang, Xiaoming Tang, Feng Ju, Meng Xiao, Tengfei Wang, Dong Wang, Lidong Yin, Lu Si, Mengxin Xu

**Affiliations:** 1 State Key Laboratory of Intelligent Construction and Health Operation and Maintenance of Deep Engineering, China University of Mining and Technology, Xuzhou 221116, China; 2 Shanghai Datun Energy Co., Ltd., Xuzhou 221611, China

**Keywords:** pure gangue slurry, pumpability, particle size distribution, water-solid ratio

## Abstract

Gangue slurry pumping backfill offers a cost-effective and environmentally sound solution for coal mine solid waste disposal. Addressing the poor pumpability of pure gangue slurry, this study applied the Talbot gradation theory to a non-cemented gangue system by designing various particle size gradations and water-solid ratios (W/S). Through tests on rheological properties, slump, spread, and bleeding rate, the optimal proportion for pumpability of pure gangue slurry (PGS) within the scope of this study was determined. Tests were conducted on rheology, slump, spread flow, and bleeding rate to determine the optimal mix proportion for pumpability. The results show that: The slurry in this study demonstrates a strong correlation with the characteristics of a Bingham fluid. Its yield stress increases significantly as the W/S decreases. At a gradation index (*n*) of 0.4, particle packing is densest, resulting in the lowest yield stress. Slump and spread flow decrease with a lower W/S. They initially increase and then decrease as the gradation index increases, with optimal fluidity observed at *n* = 0.4. Bleeding rate increases with a higher gradation index but decreases with a lower W/S. Comprehensive optimization determined the optimal mix proportion as gradation index *n* = 0.4 and W/S of 0.18. At this ratio: Yield stress = 144.25 Pa, Slump = 255 mm, Spread flow = 60.1 cm, Bleeding rate = 2.21%. This meets the pumping requirements (Slump > 180 mm, Bleeding rate < 3%). The research results provide important experimental value for the practical pipeline transportation of PGS and the reduction in pumping friction resistance.

## 1. Introduction

Coal gangue, a by-product generated during coal mining, accounts for approximately 10–25% of raw coal output [1]. Statistics indicate that China’s cumulative stockpile of coal gangue currently exceeds 7 billion tons, with annual production still increasing at a rate of 300–350 million tons [2,3]. This massive quantity occupies valuable land resources, restricting its reuse and development. Furthermore, coal gangue contains significant amounts of harmful substances and can undergo spontaneous combustion under suitable conditions, polluting air and water resources and impacting human health [4,5,6]. Underground gangue backfilling [7,8,9] is one technique for its efficient and harmless disposal. Zhang et al. [10] proposed a grouting backfilling method utilizing subsequent mining-induced voids. Essentially, this involves crushing gangue to a specific particle size gradation, mixing it with water to form a PGS, and then transporting it via filling pumps and pipelines into the voids created after coal extraction. During backfilling, the slurry must possess excellent fluidity and stability to ensure safe transportation over long distances through pipelines [11,12,13]. Compared to cement-based gangue slurries, PGS exhibit lower fluidity and poorer stability, increasing the risk of pipeline blockage [14,15]. Therefore, determining an appropriate particle gradation and water content becomes a critical issue for the pumpable backfilling of PGS.

Research on the transport performance of backfill slurries has been widely conducted, with some scholars investigating slurry transport properties by adjusting gangue particle size gradation. Jin et al. [16] classified gangue particle sizes based on fractal theory and studied the slurry’s rheological properties, finding that a higher fractal dimension indicates more fine particles, and adjusting the slurry concentration when fine particle content reaches a threshold value can achieve an optimal slurry state. Liu et al. [17] divided gangue particles into three size ranges and, by analyzing the effects of mass concentration, fly ash percentage, and aggregate gradation on slurry rheology and stability, developed a backfill slurry meeting reasonable performance indicators. Sun et al. [18] employed response surface methodology to study the rheological properties of backfill slurry under different gangue mass fractions and particle gradations, identifying the optimal gangue mass fraction and particle gradation. Other researchers have focused on optimizing the material mix proportion of backfill slurries to ensure pumpability. Qiu et al. [19] experimentally and via machine learning investigated the influence of factors like cement content, mass concentration, and fly ash-to-slag ratio on backfill slurry properties. Their findings revealed that increasing both cement content and mass concentration effectively enhances slurry stability, and the fly ash/slag ratio significantly impacts slurry performance. Zheng et al. [20], through orthogonal experiments, studied the effect of different mass concentrations of coal gangue and fly ash on paste pumpability. Their research indicated that fine gangue particles have a greater impact on material slump than coarse particles, increasing mass concentration adversely affects paste slump, and adding fly ash content improves paste pumpability. Zhang et al. [21] investigated the optimal mixing ratio of coal gangue, fly ash, and a “wind-blown sand + loess” mixture. Wang et al. [22] used a high-concentration gangue slurry rheometer to study its rheological properties, finding that both plastic viscosity and yield stress increase with rising mass concentration. Wang et al. [23] employed an intelligent torque rheometer for testing to determine the optimal mix ratio for slurry components. Liu [24] discovered that gangue particles below 0.315 mm can form a slurry with good fluidity when mixed with water, exhibiting characteristics similar to conventional fly ash mortar. Furthermore, adjusting the particle size distribution of gangue and the water-to-gangue ratio can yield better flow performance.

In summary, previous studies mainly addressed cemented or fly ash-containing slurries, while PGS remains underexplored. This study addresses backfill mining scenarios without surface protection requirements, primarily utilizing abandoned roadway spaces where backfill strength is not a critical factor. Taking rheological properties, fluidity, and stability as the key research indicators, gangue is classified into different particle size gradations using the Talbot gradation theory. Rheological tests, slump tests, and bleeding rate tests are employed to investigate the flow performance of PGS under various gradations and W/S, aiming to determine the optimal mix proportion. This study aims to determine the optimal gradation and water–solid ratio for achieving pumpable pure gangue slurry using Talbot theory. It provides an experimental basis for the pipeline transportation performance of PGS, so as to promote the large-scale and efficient disposal of coal gangue.

## 2. Materials and Methods

### 2.1. Basic Properties of Raw Materials

**Figure 1:**
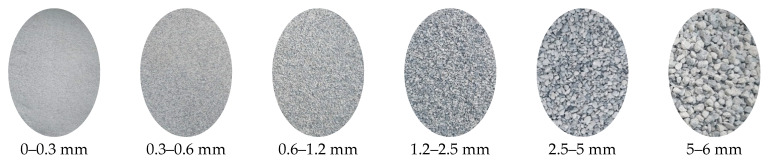
Gangue samples.

**Figure 2:**
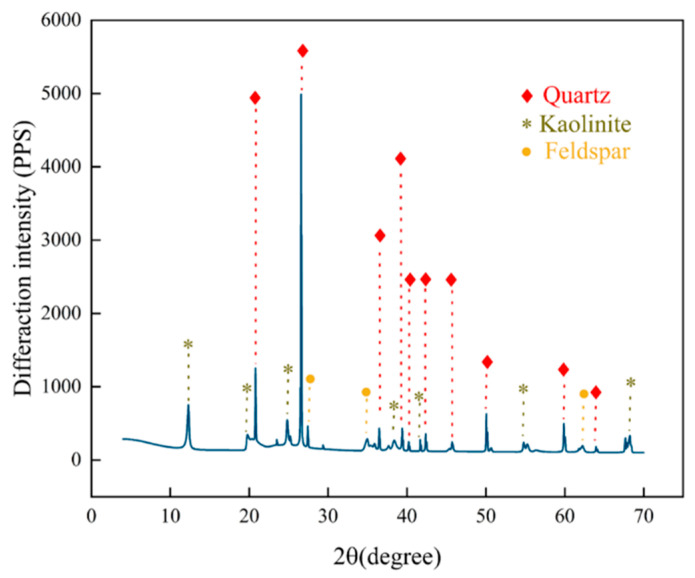
XRD spectrum of gangue.

**Figure 3:**
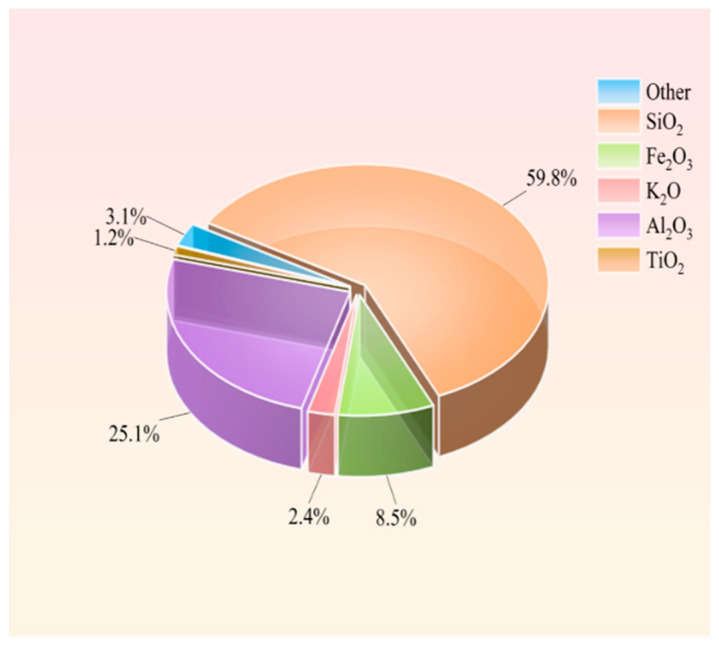
Content of each oxide by XRF/%.

The materials used in this study were gangue and water, with the gangue sourced from tunneling waste rock of Xuzhuang Coal Mine, Datu Energy Co., Ltd. in Xuzhou, Jiangsu Province, China, and the water being laboratory tap water. The gangue was crushed using a jaw crusher and sieved into six particle size fractions: (0–0.3 mm), (0.3–0.6 mm), (0.6–1.2 mm), (1.2–2.5 mm), (2.5–5 mm), and (5–6 mm), as shown in Figure 1. Subsequently, the gangue was pulverized to below 0.1 μm using a BM6Pro planetary ball mill for XRD and XRF analyses (Figure 2 and Figure 3). Figure 2 reveals that the primary mineral constituents of the gangue are quartz, kaolinite, and feldspar, classifying it as typical kaolinite-based coal gangue. Figure 3 demonstrates that the chemical composition consists predominantly of silicon dioxide (59.8%), aluminum oxide (25.1%), iron oxide (8.5%), and trace oxides of potassium (2.4%) and titanium (1.2%), with silicon dioxide exhibiting the highest proportion.

### 2.2. Talbot Gradation Theory Design Test

The Talbot [25,26] gradation theory is widely applied in the field of mineral materials and backfill materials as a gradation design methodology, refined by A.N. Talbot from Fuller’s maximum density curve theory. It primarily optimizes particle size distribution by adjusting the gradation coefficient (*n*-value) to achieve balanced engineering performance. Since PGS consists solely of gangue and water, the rational proportioning of different particle sizes plays a crucial role in determining the slurry’s flowability and stability.

According to Talbot’s gradation theory [27], the ratio *P_i_* of the mass *M_i_* of aggregate particles with particle size ≤ *d_i_* to the total mass *M_t_* in the specimen is defined by the expression:

(1) $$P_{i}=\frac{M_{i}}{M_{t}}={\left(\frac{d_{i}}{d_{max}}\right)}^{n}$$

In the equation, *d_max_* denotes the maximum particle size of the aggregate, *n* represents the Talbot gradation index, and according to Equation (1), the mass $M_{d_{1}}^{d_{2}}$ of aggregate particles with sizes ranging between *d*_1_ and *d*_2_ is expressed as:

(2) $$M_{d_{1}}^{d_{2}}=\left[{\left(\frac{d_{2}}{d_{max}}\right)}^{n}-{\left(\frac{d_{1}}{d_{max}}\right)}^{n}\right]M_{t}$$

**Figure 4:**
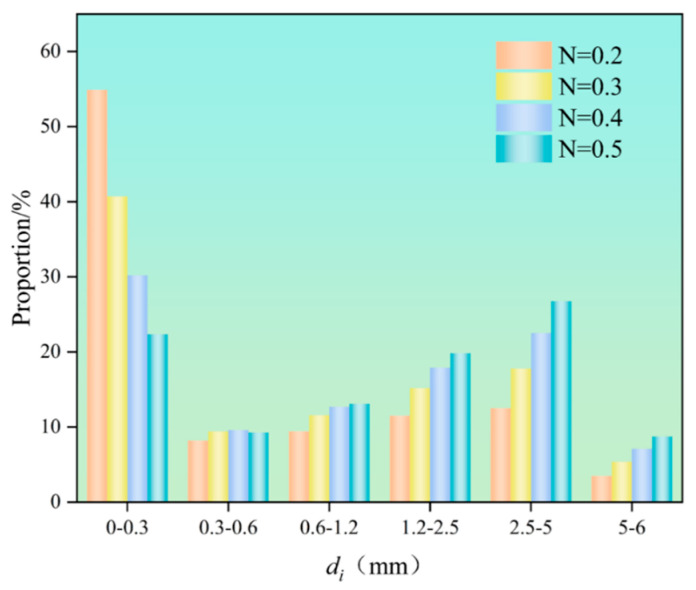
The mass ratio distribution of Talbot index aggregate particles with different gradations.

According to Equation (2), the mass percentage of aggregate particles within each size fraction under different gradation indices for every test group can be derived, as illustrated in Figure 4. It is evident that as the gradation index increases, the proportion of fine particles (0–0.3 mm) gradually decreases while the proportion of coarse particles (2.5–6 mm) progressively rises, with an equilibrium state between fine and coarse particles achieved at a gradation index of 0.4.

### 2.3. Test Methods

**Figure 5:**
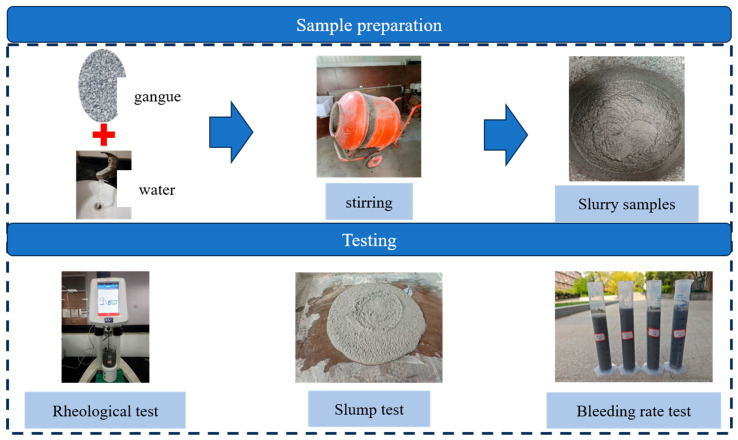
Experimental flowchart of PGS.

This study evaluated the rheological properties, flowability, and stability of PGS using a rheometer, slump cone, and graduated cylinder, with the experimental flowchart presented in Figure 5.

#### 2.3.1. Rheological Test

**Figure 6:**
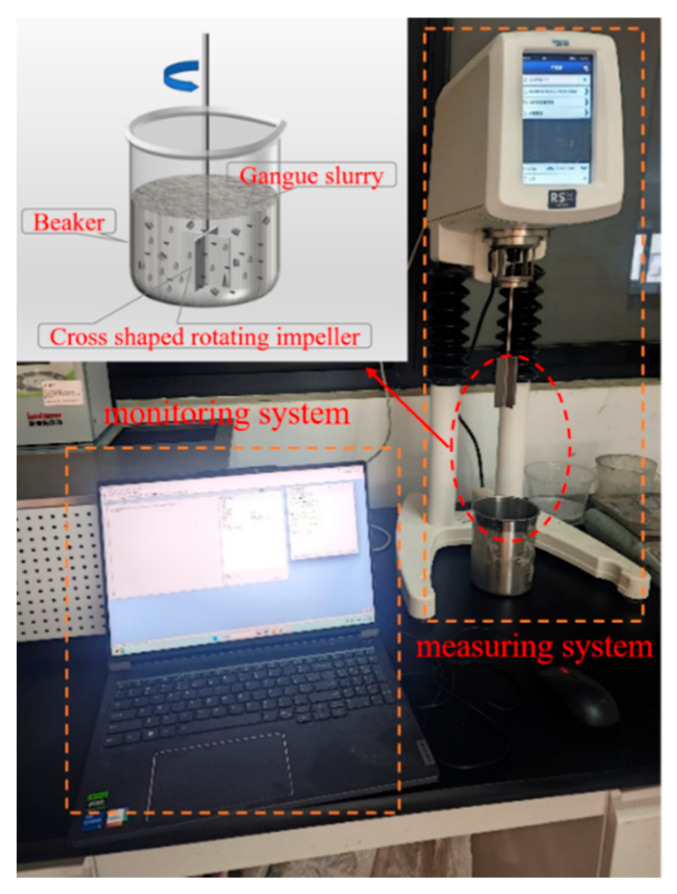
Rheological property test.

The characterization of slurry rheological properties hinges on yield stress and viscosity, where higher yield stress typically necessitates greater pumping pressure to initiate flow, whereas elevated viscosity results in reduced pumping rates. During long-distance pipeline transport, excessive yield stress may increase pumping resistance and precipitate pipe blockage, thus underscoring the need to maintain low yield stress and appropriate viscosity [28,29,30]. This study utilized a Brookfield RSX rheometer from Ametek Brookfield (Middleborough, MA, USA) equipped with a VT-80-40 vane rotor for rheological testing. The testing procedure was conducted using Rheo3000 software (1.2.1395.1), as illustrated in Figure 6. The vane rotor has a shear stress range of (0.7–420 Pa) and a viscosity range of (0.002–178) Pa·s. The tests were performed under controlled shear rate (CSR) conditions, with the shear rate increasing from 1 s^−1^ to 50 s^−1^ over a total duration of 100 s, acquiring one measurement point per second. The temperature range for the tests was maintained between −20 °C and 180 °C.

#### 2.3.2. Slump Test

**Figure 7:**
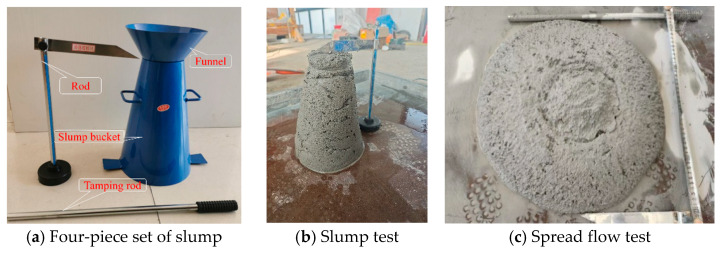
Slump test process.

The underground transportation of gangue slurry during backfill operations primarily relies on pipeline pumping, necessitating specific fluidity requirements where slump serves as a critical indicator [31,32,33]. Slump testing was conducted according to the GB/T 50080-2002 standard (Standard for test method of performance on ordinary fresh concrete) [34], as illustrated in Figure 7, using a slump cone characterized by an upper diameter of 100 mm, lower diameter of 200 mm, and height of 300 mm. The prepared PGS was poured into the cone in three successive layers, with each layer consolidated through repeated rodding to ensure uniform distribution. After filling, the cone was vertically lifted, allowing the slurry to settle; the resulting vertical difference between the cone height and the slumped slurry’s peak surface defines the slump value. Upon stabilization of slurry spreading, the radial flow distance was measured as the spread flow, reflecting the slurry’s flow behavior and frictional resistance. Empirical backfill experience indicates that a slump greater than 180 mm is generally required to meet pumping specifications.

#### 2.3.3. Bleeding Rate Test

Bleeding rate refers to the proportion of water separated from slurry due to sedimentation of solid particles under static conditions, where a higher bleeding rate compromises slurry stability [35,36,37]. During PGS backfilling, the substantial solid content accelerates particle sedimentation; consequently, excessive bleeding rate may elevate pumping resistance and increase pipe blockage risks. Based on global backfilling experience, the bleeding rate should be maintained below 3%.

This study employed graduated cylinders for bleeding rate measurement at 20 ± 2 °C ambient temperature. After recording the cylinder’s initial mass (m_1_), freshly mixed slurry was poured into the cylinder, lightly vibrated to eliminate visible air bubbles, and weighed (m_2_). The cylinder was then sealed with plastic wrap to prevent evaporation, whereupon supernatant bleed water was extracted via pipette at 30 min intervals until no water could be withdrawn for three consecutive cycles. Finally, the total bleeding quality was calculated.

The bleeding rate is calculated as follows:

(3) $$B=\frac{W_{b}}{(W/M)m_{3}}\times 100\%$$

In the formula, *B* denotes the bleeding rate (*%*), *W_b_* represents the total mass of bleed water (*g*), *W* corresponds to the mass of mixing water (*g*), *M* signifies the total mass of the mixed backfill material (*g*), and *m_3_* indicates the sample mass (*g*), where *m_3_* = *m*_2_ − *m*_1_.

## 3. Results and Analysis

### 3.1. Test Result Analysis

Testing yielded the yield stress, viscosity, slump, spread flow, and bleeding rate of PGS under gradation indices of 0.2, 0.3, 0.4, and 0.5 and W/S of 0.22, 0.20, 0.18, and 0.16, with results presented in Table 1. Yield stress and viscosity data were unobtainable for the combination of W/S 0.16, gradation 0.2, and W/S 0.18 due to shear stress exceeding instrument measurement limits. The yield stress ranged from 39.52 to 382.69 Pa, peaking at 382.69 Pa (gradation 0.5, W/S 0.18) and reaching its minimum of 39.52 Pa (gradation 0.4, W/S 0.22). Viscosity varied between 0.51 and 3.71 Pa·s, attaining its maximum of 3.71 Pa·s (gradation 0.5, W/S 0.22) and minimum of 0.51 Pa·s (gradation 0.3, W/S 0.18). Slump and spread flow spanned 30–290 mm and 21–78.5 cm, respectively, achieving maxima of 290 mm and 78.5 cm (gradation 0.4, W/S 0.22) and minima of 30 mm and 21 cm (gradation 0.2, W/S 0.16). The bleeding rate fluctuated from 0.24% to 10.31%, culminating at 10.31% (gradation 0.5, W/S 0.22) and diminishing to 0.24% (gradation 0.2, W/S 0.16). Analysis confirms only three cases (S2, S7, S11) satisfy backfill requirements.

**Figure 8:**
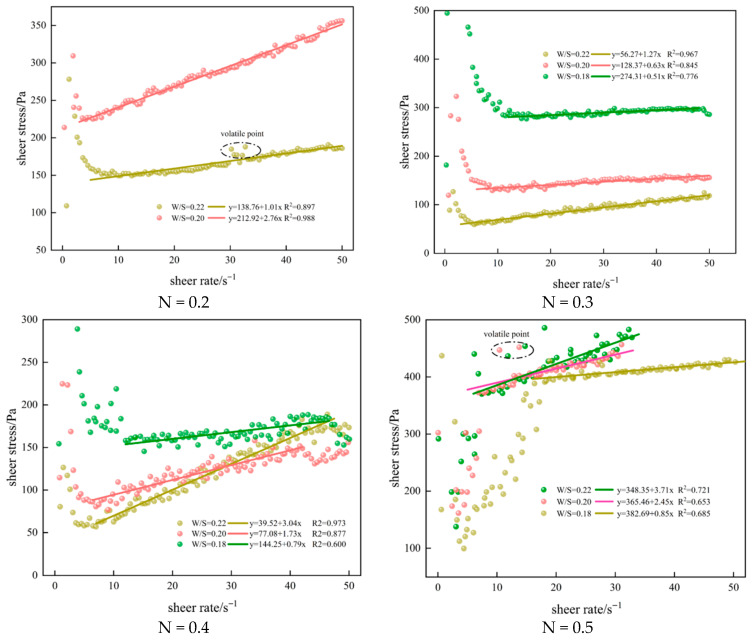
Rheological test.

The rheological curve of the slurry was fitted as shown in Figure 8, where the straight line is a linear fit used to estimate yield stress and plastic viscosity under a Bingham model for this slurry. Note where measurements stopped due to instrument limits. The figure indicates that the shear stress of the slurry increases linearly with the shear rate, with significant fluctuations observed due to the high particle content in the PGS. This effect is most pronounced at a gradation index of 0.5, where elevated coarse particle content accelerates sedimentation at low shear rates, establishing vertical concentration gradients while inter-particle collisions cause substantial additional energy dissipation, consequently generating maximum data scatter. At this gradation (*n* = 0.5), measurements ceased near shear rates of 35 s^−1^ as the shear stress reached the instrument’s maximum measurable limit. It can be seen from the figure that the rheological behavior of the PGS in this study closely matches that of a Bingham fluid, where the intercept and slope of the fitted line correspond to the slurry’s yield stress and viscosity, respectively [38].

### 3.2. The Influence of Rheological Properties Under Different Gradation and W/S

**Figure 9:**
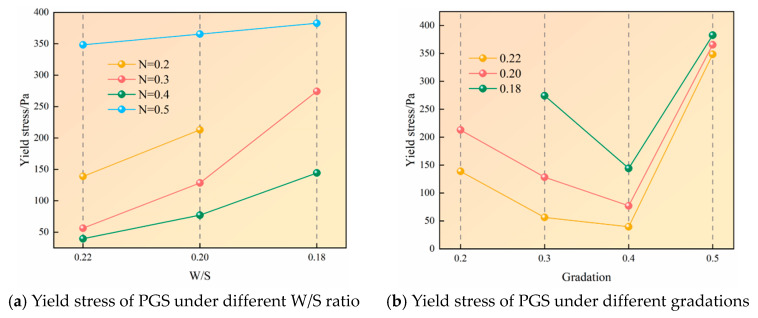
Yield stress of PGS.

Analysis of the existing data indicates that the yield stress of the slurry generally increases linearly with a decrease in the W/S, while it initially decreases and then increases with a rise in the gradation index, as shown in Figure 9. This dual-trend behavior originates from complementary mechanisms: Firstly, the high specific surface area of fine particles promotes floc formation through water-mediated interactions, creating inter-floc networks that resist external deformation. Reduced W/S intensify particle flocculation, enhancing structural resistance and consequently elevating yield stress [39,40]. Secondly, yield stress primarily stems from interparticle friction; diminished water content increases solid concentration per unit volume, reduces interparticle spacing, and amplifies interaction forces, thereby requiring greater external energy to initiate flow [41]. Concurrently, gradation adjustments modify particle packing density—optimal gradation enhances compactness, mitigates frictional resistance, and reduces yield stress.

Figure 9a demonstrates that reducing W/S from 0.22 to 0.2 at gradation 0.2 increased shear stress by 53.4%, whereas comparable reductions to 0.18 at gradations 0.3, 0.4, and 0.5 yielded increases of 387.5%, 265%, and 9.8%, respectively. Figure 9b reveals that increasing gradation from 0.2 to 0.4 at W/S 0.22 and 0.20 reduced yield stress by 71.5% and 63.8%, respectively, while at W/S 0.16, the 0.3→0.4 gradation increase caused a 47.4% reduction. Subsequent gradation elevation substantially increased yield stress by 781.5%, 374.1%, and 165.3% across descending W/S.

The underlying mechanism involves three phases: Initial gradation increase (0.2→0.4) reduces fine particle content, diminishing both floc quantity and network strength, causing rapid structural collapse under shear and significant yield stress reduction. Simultaneously, increasing (2.5–6 mm) coarse particles optimize interparticle porosity to form dense configurations that minimize frictional contacts. Gradation of 0.4 achieves coarse-to-fine equilibrium with maximal packing density and minimal friction. Further gradation increase (>0.4) disproportionately elevates coarse fraction, disrupting the floc network through sedimentation. Although deformation resistance diminishes, insufficient fines fail to fill inter-coarse voids, creating loose structures where collision resistance and friction dominate, consequently elevating yield stress anew.

### 3.3. The Influence of Fluidity Under Different Gradation and W/S

**Figure 10:**
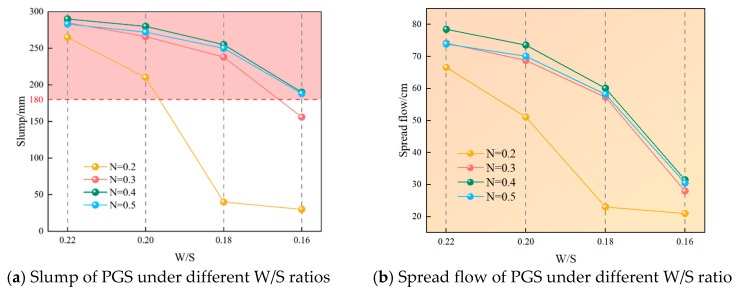
The fluidity of PGS under different W/S ratios.

**Figure 11:**
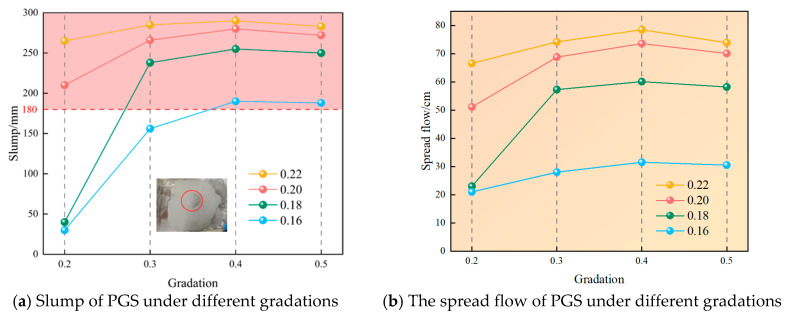
The fluidity of PGS under different gradations.

Analysis reveals that under identical particle size gradation, both slump and spread flow of PGS decrease with reduced W/S, while at fixed W/S, they initially increase then decrease as gradation index rises, as shown in Figure 10 and Figure 11. This behavior primarily stems from water’s dual lubricating functions: Firstly, fine particles (0–0.3 mm) disperse within the continuous aqueous phase to form fine-slurry coatings that suspend coarse particles (predominantly 2.5–5 mm), thereby reducing van der Waals forces and inter-coarse friction [42,43]. Reduced W/S diminish available water, increasing fine-slurry viscosity which must simultaneously overcome its own viscous resistance and lubricate coarse particle contacts, consequently impairing fluidity. Secondly, optimal gradation minimizes inter-aggregate voids, reducing water demand for void filling and liberating more water for lubrication, thus enhancing slurry flowability.

Figure 10 demonstrates that at gradations 0.3, 0.4, and 0.5, slump and spread flow decline gradually until W/S 0.18, with slump reductions of 16.5%, 12.1%, and 11.7% and spread flow decreases of 22.8%, 23.4%, and 21.2%, respectively, when ratio decreases from 0.22 to 0.18. Below 0.18, inflection points occur where slopes steepen markedly: slump drops by 34.2%, 25.4%, and 24.8%, while spread flow plunges 51.1%, 47.5%, and 47.6%, respectively. This accelerated decline originates from sufficient water above ratio 0.18 enabling fine-slurry suspension of coarse particles, whereas further reduction breaches the critical lubrication threshold where limited water is captured by fines, creating “fine-particle-hydrated/coarse-particle-dry” conditions that drastically increase friction.

At gradation 0.2 with W/S > 0.18, slump and spread flow curves exhibit steeper initial slopes than other gradations: decreasing ratio from 0.22 to 0.2 causes 20.7% slump and 23.3% spread flow reductions. Below ratio 0.2, an inflection point emerges where slopes intensify sharply, yielding 80.9% slump and 54.9% spread flow declines. Subsequent ratio reduction decelerates the descent to 25% and 8.7%, respectively. This triphasic behavior occurs because gradation 0.2 contains substantially higher fine-particle (0–0.3 mm) content, demanding more water for lubrication and forming highly viscous fine-slurry that accelerates fluidity loss and prematurely reaches the critical lubrication threshold; further water reduction only marginally increases friction thereafter.

Figure 11 reveals that increasing gradation from 0.2 to 0.3 elevates slump by 7.5%, 26.7%, 495%, and 420% across descending W/S, with corresponding spread flow increases of 11.4%, 34.6%, 149.1%, and 33.3%. Notably, this transition reduces (0–0.3 mm) particle fraction from 54.9% to 40.7% while increasing (2.5–6 mm) coarse fraction from 16% to 23.13%, enhancing coarse-fine particle continuity. Fine particles begin filling the skeletal framework formed by coarse aggregates, intensifying interparticle embedding and reducing porosity. This simultaneously diminishes interparticle friction/collision and develops free water channels that release additional lubricating water, thereby enhancing slurry fluidity [44].

Further gradation increase to 0.4 reduces fine particles (0–0.3 mm) from 40.7% to 30.2% while elevating coarse fraction (2.5–5 mm) from 23.13% to 29.6%, achieving coarse-to-fine equilibrium. This optimal state yields minimal porosity, lowest interparticle friction, minimum yield stress, and sufficient water lubrication, consequently maximizing slump and spread flow. Subsequent gradation increase beyond 0.4 further reduces fines to 22.36% and increases coarse fraction to 35.47%, disrupting the equilibrium. Here, fine slurry becomes insufficient to suspend coarse particles, triggering gravitational settling where inter-coarse friction dominates, ultimately reducing slump and spread flow.

### 3.4. The Influence of Stability Under Different Gradation and W/S

**Figure 12:**
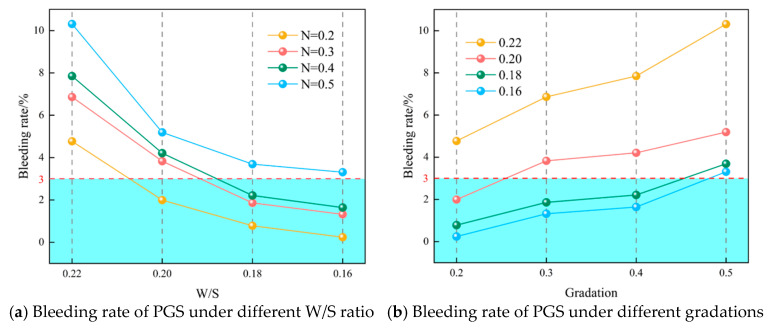
Bleeding rate of PGS.

Analysis indicates that at identical gradation indices, slurry bleeding rate decreases linearly with reduced W/S, while at fixed W/S, it increases linearly with rising gradation index, as shown in Figure 12. As established previously, fine particles form flocs through water-mediated interactions, creating floc networks that entrap free water via a “water-locking effect.” Reduced W/S diminish free water while increasing floc formation, enhancing water retention and consequently decreasing bleeding rate to improve slurry stability [45]. Conversely, higher gradation indices increase coarse particle content—their smaller specific surface area inhibits floc formation, while greater mass accelerates sedimentation, creating favorable conditions for water separation.

Comprehensive analysis confirms that at a gradation index *n* = 0.4, optimal particle continuity allows fine particles to sufficiently embed within the coarse-particle skeletal structure, minimizing interparticle friction while ensuring adequate water lubrication. This configuration yields the lowest slurry yield stress and maximizes slump/spread flow. Concurrently, at W/S = 0.18, bleeding rate complies with pumping specifications. Integrated evaluation ultimately determines *n* = 0.4 with W/S = 0.18 as the optimal mixture proportion.

Figure 12a demonstrates that reducing W/S from 0.22 to 0.16 decreases bleeding rate by 94.9%, 80.8%, 79.1%, and 67.9% across ascending gradations. Figure 12b reveals that increasing gradation elevates bleeding rate by 116.1%, 160.8%, 373.1%, and 1279.2%, respectively, across descending W/S. This escalation occurs because higher gradation reduces fine particle proportion, decreasing specific surface area and floc formation. Consequently, less free water is entrapped, diminishing the fine slurry’s capacity to suspend coarse particles and accelerating sedimentation, ultimately compromising slurry stability.

## 4. Conclusions

This study has designed 16 distinct mixture proportions of PGS through varying gradation indices and W/S, with comprehensive analysis conducted across rheological properties, flow characteristics, and stability performance, yielding the following principal conclusions:

1. In this study, the shear stress of the PGS increases with the shear rate, demonstrating good agreement with the Bingham rheological model. As W/S decreases, heightened slurry concentration intensifies particle flocculation while reducing free water availability, diminishing lubrication; consequently, yield stress rises and fluidity declines, though stability improves. Lower gradation indices with abundant fine particles necessitate excessive water, rendering them unsuitable for high-concentration backfill applications.
2. Increasing gradation reduces fine particle content, diminishing free water entrapment capacity and weakening the floc network structure while improving particle continuity; consequently, PGS exhibits an initial decrease followed by an increase in yield stress, with fluidity first enhancing then declining. At gradation *n* = 0.4, coarse-to-fine equilibrium achieves minimal yield stress and optimal fluidity, though bleeding rate maintains a persistent upward trajectory.
3. The optimal mixture proportion for PGS is established at gradation *n* = 0.4 combined with W/S 0.18, where balanced coarse-to-fine particle distribution enables thorough occupation of the coarse-particle skeletal framework by fines, achieving minimal porosity while comprehensively satisfying all pumping specifications. This study provides an important experimental basis for the selection of crushing parameters and mixture proportioning in subsequent industrial-scale mine backfill systems.

The aforementioned study confirms the feasibility of using PGS as a backfill material, providing an experimental foundation for subsequent engineering applications.

In the future, we will systematically analyze factors influencing the transport performance of the slurry—such as particle morphology, mineral composition, and packing density under various gradations—as well as conduct loop-pipe tests and numerical simulations based on factors like pipeline wear and pressure loss under long-distance transport conditions, ultimately establishing a comprehensive theory for the pipeline pumping and backfilling of PGS.

## Figures and Tables

**Table 1:** Test results of rheological property, fluidity, and stability of PGS.

Numb	Gradation	W/S	Yield Stress/Pa	Viscosity/Pa·s	Slump/mm	Spread Flow/cm	Bleeding Rate/%
S1	0.2	0.22	138.76	1.01	265	66.6	4.77
S2	0.2	0.20	212.92	2.76	210	51.1	1.99
S3	0.2	0.18	/	/	40	23	0.78
S4	0.2	0.16	/	/	30	21	0.24
S5	0.3	0.22	56.27	1.27	285	74.2	6.86
S6	0.3	0.20	128.37	0.63	266	68.8	3.83
S7	0.3	0.18	274.31	0.51	238	57.3	1.86
S8	0.3	0.16	/	/	156	28	1.32
S9	0.4	0.22	39.52	3.04	290	78.5	7.85
S10	0.4	0.20	77.08	1.73	280	73.6	4.21
S11	0.4	0.18	144.25	0.79	255	60.1	2.21
S12	0.4	0.16	/	/	190	31.5	1.64
S13	0.5	0.22	348.35	3.71	283	73.9	10.31
S14	0.5	0.20	365.46	2.45	272	70.1	5.19
S15	0.5	0.18	382.69	0.85	250	58.2	3.69
S16	0.5	0.16	/	/	188	30.5	3.31

Note: /—exceeded instrument limits, no value reported.

## Data Availability

The original contributions presented in this study are included in the article. Further inquiries can be directed to the corresponding author.
